# Effectiveness after 5 years of the who hand hygiene promotion strategy to reduce health-care-associated infections at Hung Vuong Hospital, Vietnam

**DOI:** 10.1186/2047-2994-4-S1-O20

**Published:** 2015-06-16

**Authors:** PT Hang, TTT Hang, DPP Anh, W Zingg, D Pittet

**Affiliations:** 1 Infection control, Hung Vuong Hospital, Ho Chi Minh, Vietnam; 2 University of Geneva Hospitals, Geneva, Switzerland

## Introduction

Hung Vuong hospital (HVH) is a 900 bed maternity hospital in Ho Chi Minh City, Vietnam, with at least 40’000 deliveries per year. In 2009, at Hung Vuong hospital (HVH), hand hygiene compliance among healthcare staff was low (8%) while the rates of neonatal hospital-acquired infections (HAIs) were quite high (15.7%). There was the need for promoting hand hygiene using the WHO strategy to improve staff compliance and possibly reduce HAIs.

## Objectives

To evaluate the improvement of hand hygiene compliance and its possible impact on HAIs at HVH.

## Methods

Hand hygiene promotion started in 2010 using the WHO multimodal strategy available from the WHO website. Following pilot phases between 2008 and 2009, surveys to assess HHC and monitor HAI rates have been conducted since 2010. The total amount of alcohol-based handrub used was monitored in parallel.

## Results

We conducted 12 surveys between 2010 and 2014 monitoring a total of 34,415 opportunities for hand hygiene during 2,688 observation sessions (i.e. for around 714 hours). The overall hospital-wide compliance with hand hygiene practices increased significantly from 8% in 2010 to 52% in 2014 (p<0.0001).

The overall consumption of alcohol-based handrub increased in parallel from 2.2L (2010) to 11.7L per 1000 patient-days (2014). The adults’ HAIs rates were reduced from 0.63 (2010) to 0.45 (2014) per 100 admissions (p=0.002), the neonatal HAIs rates were reduced from 16.1 (2010) to 5.18 per 100 admissions (2014) (p<0.0001). The neonatal mortality was reduced from 2.35 (2010) to 1.00 (2014) per 100 admissions (p=0.015).

## Conclusion

The hand hygiene promotion strategy was associated with marked improvement in staff compliance, reduction in HAIs and in overall mortality among neonates.

## Disclosure of interest

None declared.

